# Higher education students’ aspirations for their post-university lives: evidence from six European nations

**DOI:** 10.1080/14733285.2021.1934403

**Published:** 2021-06-22

**Authors:** Rachel Brooks, Achala Gupta, Sazana Jayadeva

**Affiliations:** Department of Sociology, University of Surrey, Guildford, UK

**Keywords:** Aspiration, higher education, students, Europe, place

## Abstract

While there is now a relatively large literature on young people's aspirations with respect to their transitions from compulsory schooling, the body of work on the aspirations of those within higher education is rather less well-developed. This article draws on data from undergraduate students in six European countries to explore their hopes for their post-university lives. It demonstrates that although aspirations for employment were discussed most frequently, non-economic plans and desires were also important. Moreover, despite significant commonalities across the six nations, aspirations were also differentiated, to some extent at least, by national context, institutional setting and subject of study.

## Introduction

This article draws on data from six European countries to explore higher education (HE) students’ aspirations for their lives post-graduation. It considers the extent to which commonalities are evident in students’ views, and also the extent to which they appear to be patterned by their national context, the institution they attend, and their subject of study.

There is now a large literature on young people's aspirations with respect to entering higher education. This has typically problematised policy approaches that see aspiration-raising as a means to improve the participation of non-traditional groups, arguing that, on the whole, such groups tend to have aspirations that are very similar to those from ‘traditional’ backgrounds. Such studies have argued, instead, that it is differential attainment and expectations that explain their under-representation, both of which are significantly shaped by the perspectives of the adults around them (e.g. Croll and Attwood 2013; Harrison and Waller 2018). The body of work on the aspirations of those *within HE* is rather less well-developed (although see important exceptions by, for example, Armstrong and Hamilton 2013; Bathmaker et al. 2016; Brooks 2006; Stevens, Armstrong, and Arum 2008).

The article contributes to the limited scholarship that has focussed specifically on HE students’ aspirations by examining the hopes of young people enrolled in degree-level study across Europe. It understands aspirations as being comprised of two dimensions, which are often held in tension – what Appadurai (2013) calls the ‘ethics of possibility’ (which relates to hopes and imaginative possibilities) and the ‘ethics of probability’ (which foregrounds, instead, anticipated constraints). Thus, we discuss explicitly our participants’ hopes and also some of the ways in which they believed it may be difficult for such hopes to be realised. Our comparative approach – drawing on data from Denmark, England, Germany, Ireland, Poland and Spain – allows us to tease out patterns across the continent, and contribute to debates about the degree of convergence of higher education students’ perspectives and experiences across the European Higher Education Area.

## Background

In a speech given on 9th July 2020, Gavin Williamson, the English Secretary of State for Education, in outlining reforms for post-compulsory education, asserted that ‘We must never forget that the purpose of education is to give people the skills they need to get a good and meaningful job’ (FE Week 2020, n.p.). While this comment was roundly criticised by many of those working in education for its overly narrow perspective, it is broadly representative of the assumptions that appear to underpin many HE reforms introduced across Europe over the past decade. These have included: obliging students to move more quickly through their studies so that they are able to enter the labour market sooner; encouraging employers to have a more direct input into curricula and sit on the governing boards of universities; highlighting the likely financial returns of specific degree programmes, to inform student decision-making; incentivising students to take up places on science, technology, engineering and maths (STEM) courses, on the basis of ostensible employer demand; and reducing the number of places available in subject areas that are deemed not to serve well the labour market (see, for example, Degn and Sørensen 2015; OfS 2020; Sarauw and Madsen 2020; Walsh and Loxley 2015). Moreover, students have also often been positioned discursively as first-and-foremost ‘future workers’ within many European HE policy documents (Brooks 2018b, 2021).

To a large extent, the academic literature on students’ aspirations and post-HE trajectories has mirrored this policy context. There have been a small number of studies that have focussed on, for example, students’ aspirations for further study – and how these may be affected by: the teaching they have received (Hanson, Paulsen, and Pascarella 2016); their broader undergraduate experiences (Armstrong and Hamilton 2013; Bathmaker et al. 2016; Brooks 2006; Brooks and Everett 2008; Stevens, Armstrong, and Arum 2008); and their sense of civic duty (Power et al. 2013). The vast majority, however, have focussed on aspirations for employment, and the extent to which they are realised in contemporary labour markets. This has demonstrated that while HE continues to play an important role in expanding horizons and facilitating access to well-regarded jobs (Bathmaker et al. 2016), in a range of different national contexts, transitions into the labour market are – even for graduates – often strongly patterned by social characteristics. Although scholars have focussed on the impact of both gender and ethnicity (e.g. Algan et al. 2010; Bunel and Guironnet 2017), the most substantial body of work addresses the impact of social class. Research has shown how graduates from lower socio-economic groups often have worse employment outcomes than their more privileged peers (e.g. Britton et al. 2016), and are over-represented in the public sector and caring occupations, which tend to have lower average wages (Christie et al. 2018). These differences often pertain to students who have graduated from elite institutions (Christie et al. 2018; Hurst 2018). Explanations for these enduring differences typically focus on the differential resources to which students have access, which can affect what they do whilst at university and immediately afterwards, and the practices of graduate employers.

Hurst (2018) analysed the work and/or study destinations of those who graduated from American liberal arts colleges between 2012 and 2014. She showed that, despite all of her survey respondents having attended selective institutions – which may ordinarily be seen as a mark of success – there were significant differences by social class in both employment outcomes and progression to further study. In contrast to their peers from lower socio-economic status backgrounds, those from richer families had typically been able to participate in costly extra-curricular activities during university, through which they had developed social networks that helped them to find graduate jobs, and had been able to take up unpaid internships to gain valuable experience in the workplace. They were also able to draw on advice from family members about which university courses were the most likely to lead to well-paid employment. Indeed, there were clear differences in subject studied amongst her respondents, which had a significant impact on the choices open to them on graduation. In general, she found that those from upper class backgrounds were most likely to have studied STEM subjects (which often led to very well-paid jobs), while those from working class backgrounds were more typically found in applied subjects such as business, with less obvious pathways to well-remunerated employment. Similar findings about the impact of class on subject choice has been found in other parts of the world – see, for example, Codiroli McMaster (2019) and Davies et al. (2013). Furthermore, on graduation, the more privileged students in Hurst's study did not need to worry about repaying student loans – thus giving them more flexibility to undertake unpaid work and other opportunities. Such differentials in economic, social and cultural capital can be exacerbated, some scholars have suggested, by the different attitudes to the labour market of students from particular class backgrounds. While middle class students often taken a highly strategic approach to seeking employment – by, for example, exploiting their own social networks and those of their parents– their counterparts from lower socio-economic status backgrounds tend to feel less happy using family connections in this way, and believe more strongly that they should rely on their own educational qualifications and pursue only opportunities that are advertised formally (e.g. Burke 2016; Lehmann 2019).

With respect to graduate employers, studies have shown how, despite an ostensible commitment to recruiting diverse populations, practices tend to favour those with considerable familial capital (and who have often attended elite institutions). Rivera's (2015) US ethnography in three sought-after graduate professions – law, consulting and banking – demonstrated that the organisations in her study targeted only those attending high status institutions (in which those from privileged backgrounds were typically over-represented) and looked for individuals with suitable ‘polish’ with whom the recruiters believed there would be a good ‘cultural fit’. This had the effect, Rivera argues, of excluding those from poorer families who had not had the time or money to be able to take advantage of the various extra-curricular pursuits that helped their richer counterparts establish cultural connections, nor exhibited the social confidence that was seen as a key sign of ‘polish’. Similar arguments about recruitment practices and workplace norms have been made with respect to the UK (e.g. Friedman and Laurison 2019). Indeed, Ingram and Allen (2019) have shown how classed expectations of the ‘ideal’ graduate infuse even marketing materials, with employers appearing to ‘fall into the trap of assuming that displays of cultural capital evidence knowledge, skills and personal traits’ (p.737).

It is not always the case, however, that students’ social characteristics determine their employment outcomes. There is evidence that, in some circumstances, the institution attended can be a stronger predictor of a student's job prospects. Bathmaker et al.'s (2016) research in two UK HE institutions (HEIs) in the same geographical area but of different statuses indicated that those who attended the more prestigious institution had a strong advantage when entering the labour market, irrespective of social background. They explain this in terms of employer practices that favour those who have attended elite universities. Other UK-based research has also shown a strong correlation between university attended and earnings level (e.g. Wakeling and Savage 2015).

While engaging with this body of work on the aspirations of HE students – particularly in relation to post-graduation employment – this paper also speaks to debates about the extent to which HE systems across Europe have converged. Some scholars have argued that, through initiatives such as the Bologna Process, the creation of a European Higher Education Area and the impact of neo-liberalism more generally, students’ lives and perspectives have become increasingly similar across the continent. Moutsios (2013), for example, has contended that European students have become consumer-like in their orientations, while Sarauw and Madsen (2020) have highlighted the pressures on students in various European states to move through their studies quickly, with a primary focus on labour market entry, rather than the here-and-now of studying and intellectual engagement. Other researchers have, however, suggested that, despite these various initiatives and policies, national differences endure and the European space remains differentiated, at least as far as understandings of students are concerned (e.g. Brooks 2018a, 2021). Indeed, focussing specifically on aspirations, Power et al. (2013) have revealed significant differences between graduates from an elite HE institution (HEI) in the UK, whose future plans centred largely on developing their own careers, and their peers at a comparable French institution who placed more emphasis on their social and civic duties. We are attentive to this debate (about the relative influence of national differences) in the rest of the article, exploring the extent to which different national contexts appeared to shape the aspirations of the HE students to whom we spoke. Unlike some of the literature discussed above, we also foreground students’ own perspectives and aspirations, as they provide an important insight into how post-graduation lives are understood.

## Methodology

This paper draws on data collected in 2017–18 through focus group discussions with 295 students in six European countries: Denmark, England, Germany, Ireland, Poland and Spain. These nations were chosen to provide diversity with respect to: welfare regime (Esping-Anderson 1990); relationship to the European Union; means of funding HE; and the extent of any financial support provided to students (see Table 1 for details). Fieldwork was conducted in three HEIs in each country, chosen to represent the diversity of the respective national HE sector. For example, in Spain, we conducted focus groups in one private university and two public institutions; in Ireland, in one institute of technology and two universities of different statuses; and in England, three universities that occupy very different market positions. Focus groups were conducted with domestic students (rather than those paying international fees), all of whom were enrolled on an undergraduate degree programme. We aimed to include students from a wide range of disciplines, to reflect the spread of subjects taught at the various institutions. However, largely because of logistical factors, students from arts and social science programmes were over-represented (see Table 2 for details). Within the sample, we aimed to maintain diversity by including students with a variety of different social characteristics (e.g. by gender, ethnicity, age group and socio-economic status). About 39 per cent of our participants were the first in their family to attend HE, and nearly 17 per cent were from a working-class background (here we used parents’ occupation as a proxy for social class^1^) (see Table 2).

**Table 1.** Characteristics of the countries involved in the research.

Country	Welfare regime	Accession to the EU	Tuition fees for full-time undergraduates in public universities (2018/19)	Student support for full-time undergraduates (2018/19) – with amounts per annum^a^
Denmark	Social democratic	1973	No tuition fees	c. 89 per cent receive needs-based grants (average of €9810); loans available to those entitled to state grant
England	Liberal	1973 (left in 2020)	Fees typically €9998 per year, paid by all students	No grants; income-contingent loans available to all for tuition; needs-based loan for maintenance costs
Germany	Corporatist	1952	No tuition fees; in 10 Länder, small administrative fee of up to €70 paid	c.22 per cent of students receive need-based grants (average of €5568 – includes integrated loan)
Ireland	Catholic corporatist	1973	‘Student contribution’ of €3000 per year paid by c.57 per cent of students	c.43 per cent of students receive need-based grants (average of €4600); no loans available
Poland	Post-Communist	2004	No tuition fees; one-off administrative fee of c.€50	c.15 per cent of students receive need-based grants (€1239) and 7 per cent merit-based grants (average €1108); loans available to those on lower incomes
Spain	Mediterranean/ sub-protective	1986	Tuition fees paid by c.70 per cent of students; average amount of €1081 per year	c.28 per cent of students receive need-based grants (average of €2166); no loans available

^a^ Source: European Commission/EACEA/Eurydice (2018).

**Table 2.** Social characteristics of student-participants across countries.

	Denmark	England	Germany	Ireland	Poland	Spain	Total	Proportion
	*N* = 42	*N* = 52	*N* = 49	*N* = 51	*N* = 45	*N* = 56	*N* = 295	100
**Gender**								
Male	15	11	14	10	15	26	91	30.8
Female	27	40	34	41	30	28	200	67.8
Others	0	1	1	0	0	1	3	1.0
Did not say	0	0	0	0	0	1	1	0.3
**Ethnicity**								
Minority ethnic^a^	6	8	1	8	0	3	26	8.8
Non-minority ethnic	35	42	48	40	27	46	238	80.7
Did not say	1	2	0	3	18	7	31	10.5
**Age group**								
<22 years	9	46	18	27	24	40	164	55.6
22–24 years	25	4	15	18	20	14	96	32.5
25–29 years	5	1	13	1	1	1	22	7.5
30 years and older	2	1	3	4	0	1	11	3.7
Did not say	1	0	0	1	0	0	2	0.7
**Subject**								
Arts and humanities	6	6	2	5	4	7	30	10.2
Social sciences	13	34	45	30	27	42	191	64.7
STEM	14	7	2	15	11	7	56	19.0
Inter-disciplinary	9	5	0	1	3	0	18	6.1
**At least one parent with HE experience**								
No	12	19	28	20	15	21	115	39.0
Yes	26	29	20	30	30	35	170	57.6
Did not say	4	4	1	1	0	0	10	3.4
**Social class**								
Middle/Upper class	33	45	37	33	35	44	227	76.9
Working class	7	6	10	11	7	9	50	16.9
Unclear	2	1	2	7	3	3	18	6.1

^a^ As the number of students from minority ethnic backgrounds was so small, we have grouped them together for the purposes of this table.

The focus groups (each lasting about 90 minutes and comprising, on average 5–6 students from similar disciplinary backgrounds) were conducted in English in Denmark, England and Ireland, and in the national language in Germany, Poland and Spain, and all were fully transcribed. Those not conducted in English were translated^2^ prior to analysis. Students were asked open-ended questions about a variety of topics such as their reasons for attending HE, what they considered to be the purpose of education, and what they thought it meant to be a student in their particular societal context. In addition, their views about some common ways in which students are understood within the academic literature and in everyday discourse – such as future workers, consumers, political actors and dedicated learners – were sought. They were also asked to make plasticine models to represent how they thought about their own identity as a student and how they believed they were understood by other social actors, and to respond to two extracts (from newspaper articles and policy documents in their country) that discussed students. The focus group transcripts were analysed using both deductive and inductive approaches, the former informed by previous work on conceptualisations of students (see Brooks 2018c for details).

The questions and the prompts students were given elicited – in various and significant ways – data about their aspirations for their post-HE lives. Given the relatively limited literature on students’ own perceptions about where they hoped and believed their HE experiences would lead, this constituted a key focus of the inductive part of our analysis. In the rest of the article we outline some of the key themes that emerged from this process. Although it would not be appropriate, given our use of focus groups, to provide information about the number of individual students who made particular comments, the themes explored below were all relatively common across the dataset (or relevant parts of it).

## Varied aspirations: individual and collective

When students spoke of their aspirations for their life after HE, and where they hoped their degree would lead, their responses were varied. In contrast to the majority of the literature discussed above, many participants focussed on non-economic factors. Some, for example, hoped that through their degree programme they would develop intellectually, gaining a more profound understanding of the subject they were studying. For some students, this was linked explicitly to broader processes of personal development. The following quotations are illustrative:

In my opinion, it's always worth having a diploma. It enables our development, not only having this diploma but moving on, going further. (Polish focus group)

I did it because I wanted, say like I wanted to further my knowledge on certain subjects and like I wanted to see the world with what I was interested in … (Irish focus group)

I see university […] as a process of cultivation and of personal improvement, and that is what I wanted to come and do here, to learn … (Spanish focus group)

While students from all countries in our sample placed emphasis on such plans, differences were evident in relation to the extent to which students framed their aspirations as part of a collective endeavour. In Denmark, Germany and Poland, in particular, many participants outlined hopes, not just for themselves, but for their wider community; they saw university as a time to develop the knowledge and skills that would help further national development.

Somehow it's all about progress … it's about advancing different subjects in order to widen our knowledge or to refute old findings and thus always … Yes it's basically about getting closer to ‘true knowledge’, perhaps to make work easier for people in the way that we build new things and how you design new things, so by planning roads in such a way that people are happier and that they are more in harmony with nature, that the economy runs more smoothly. (German focus group)

We’re such a small country, we have to do well … we’re such a small people [population] so we have to do better because there are so many people around the world who do better than us. So we have to work even harder to compete with them. (Danish focus group)

The national differences documented here reflect to some extent the findings of Power et al.'s (2013) comparison of the aspirations and orientations of English and French graduates from elite institutions (the University of Oxford and Sciences Po, respectively). They argue that while the former portrayed their futures as projects of individual self-fulfilment, moving from job to job, their French counterparts placed more emphasis on their responsibility and allegiance to their nation-state – and the contribution they would make to this in the future. While Power et al. explain these differences in terms of the national education the students had received over the course of their lives, our data suggest that students’ views may also have been informed by the messages about the purpose of HE embedded in national policies. It is interesting to note that the three countries where students more commonly articulated these collective aspirations were the ones where they were not required to pay themselves for their HE (and, in the case of Denmark, received a grant to cover their living expenses throughout the whole of their degree) (See Table 1). In such nations, because of these funding arrangements, the purpose of HE is still sometimes understood as a public good.

Nevertheless, despite this emphasis on various non-economic outcomes, it was also the case – across the six nations – that progression to employment was the aspiration that was discussed at most length in the focus groups. For many of our participants, securing a ‘good’ job (however that was defined) was a key objective of their time within HE. In many ways, this emphasis on employment reflects both the centrality of ‘future worker’ discourse in HE policy and the literature on post-university aspirations. Given the significance of employment-related concerns to our participants’ aspirations for their post-HE lives, the remainder of the article focuses specifically on this.

## Centrality of aspirations for employment: students as investors or insurers?

A large majority of students across all six countries spoke about obtaining a university degree as a crucial step towards future employment. Moreover, they believed that because of commonly-held beliefs that HE is a path to sought-after careers, members of society saw them as ‘successful’ and ‘accomplished’ through having secured a university place. Success in this context was seen as having ‘better employment prospects’ – indeed, within the focus groups, many students made plasticine models of arrows, stairs, or a compass to signify that their degree would provide an important pathway to their chosen field of work. There were, however, notable differences in how HE was conceptualised in relation to this particular aspiration. Some students saw a degree very much as a clear route to their desired employment – in fields such as journalism, medicine and law – while others viewed it offering both the time and space to explore a variety of work-related options and to enable them to reach a decision about what they wanted to do with the rest of their lives. These two contrasting perspectives are evident in these quotations:

I saw it very clearly, I finished my A levels and when I finished, I knew I would like to go on to study journalism … so I decided that the best way to do the degree and to work as a journalist or in the media, had to be via university. (Spanish focus group)

I just wanted to go to university, partially because I didn't know what I wanted to do, like career-wise, so I thought it would like help out a bit. And I was also, I did sociology at A Level and I enjoyed it, so I just kept, wanted to carry on. (English focus group)

In Germany, some participants mentioned that they had considered a more explicitly vocational route, instead of HE, but had ultimately chosen to go to university because they believed it left one's employment options open for longer.

Differences between students were also evident in the extent to which they positioned themselves as either ‘investors’ or ‘insurers’ with respect to their employment aspirations. Writing in relation to the UK in particular, Harrison (2019) has argued that over recent years the social and economic risks attached to attending university have been refigured. Because of the increasing diversity of the student body, he contends there is now less social risk associated with embarking on a degree for those from less privileged families, while as a result of the decline in the number of jobs available for those completing compulsory education, staying on to HE has become less economically risky. As a result, Harrison suggests that students are increasingly ‘insuring’ against the poor employment prospects of non-graduates, rather than assuming that an ‘investment’ in HE will lead to high status professional employment. Within our sample, there was evidence of both framings, but viewing oneself as an ‘insurer’ was common. In one of the Irish focus groups, for example, one participant commented: ‘[W]hat [is] taught nowadays is that you need a degree to get most jobs, anything other than low paying jobs you know, as far I was educated anyway when I was at school’. Here, we see students’ aspirations couched largely in terms of avoiding the low-paying work to which they believed they would be confined if they had not embarked upon a degree. Such framings were particularly evident amongst our Spanish students who often spoke about university education as a crucial means of avoiding unemployment. As we have discussed elsewhere (Brooks and Abrahams 2020; Jayadeva et al. 2020), compared to students in other countries, joblessness was the major concern for the Spanish students in our sample – reflecting broader economic conditions. At the time of data collection, the unemployment rate among recent tertiary graduates in Spain was 22.1 per cent, compared with 12.2 per cent in Denmark, 11.6 per cent in the UK, 11.1 per cent in Poland, 10.5 per cent in Ireland, 5.7 per cent in Germany (and 14.5 per cent across the EU as a whole) (Eurostat 2019). In this context, it is perhaps unsurprising that university was seen by many of our Spanish participants as an ‘insurance’ against poor labour market outcomes rather than an ‘investment’ in aspirations for high-paying professional employment.

## Impact of institution on confidence at realising aspirations

A further theme across all six countries was a belief that the ability to realise one's aspirations was closely linked to the institution attended. Indeed, many participants highlighted the various ways in which they believed their likely employment outcomes would be mediated by societal assumptions about the status of their institution. In general, students from all types of HEI and across the various countries in the sample believed that society (including employers) regarded graduates from higher-status universities as ‘more knowledgeable’, ‘super-intelligent’, and ‘superior’. This was, however, played out in rather different ways from country to country, depending on the structure of the national HE system. In Spain, for example, students attending the private university in our sample held such views, indicating that society believes that they may be advantaged in the labour market when compared to their peers at public universities. (They did not necessarily share this view themselves, though.) In Denmark, institutional differentiation was played out rather differently. Indeed, a large number of students at an HEI that tended to employ more unusual pedagogical approaches believed that their degrees were not seen by society as equivalent to those from institutions that taught in a more traditional way. They claimed:

Students here from [this institution] aren't the most well seen upon students in society, some people think that we are not specialised enough […] that we can't be used for anything […] lots of people tend to think that we’re just getting educated to go out to be […] unemployed.

Here, the students clearly did not agree with such sentiments, but suggested they may nevertheless have a tangible effect on their ability to realise their aspirations. Moreover, students from a Danish university of applied sciences made reference to how such institutions were seen as inferior to ‘traditional’ universities. According to these students, studying for a degree at a university of applied sciences was seen as easier and less work than attending a university, and leading to less well-paid jobs than a degree from a university. They said that there was also a perception that students attending ‘traditional’ universities were more intelligent than those attending universities of applied science.

In England and Ireland, students tended to differentiate between older and newer institutions, believing that transitions into professional employment would be much easier from the former. For example, focus group participants from the mid-ranking Irish university in our sample described how, despite the strengths of their degree programme, they believed that their skills and knowledge were not obvious to employers, and thus they would have to expend considerable effort to demonstrate these, in ways that would not be required of students who had attended more elite institutions: ‘ … it has the best like teaching, it has the best staff, and [yet] we constantly need to prove ourselves’. In England, similar comments were made. In the following extract, students pointed to what they believed was a significant difference in starting salaries by institution, irrespective of the particular skills graduates brought to their roles:

I feel like they’re more willing to pay them [students from older, more prestigious universities] a certain amount compared to students from other unis … even though we’ve learnt the same thing and it's [meant to be] all about your abilities of what you can bring. (English focus group, mid-ranking English HEI).

In these data, we see how students believe that labour market opportunities are shaped by societal assumptions about institution attended. Moreover, in some cases, these perceptions appeared to have affected their confidence at being able to realise their aspirations (although rarely having any discernible impact on the aspirations themselves). While the quotations provided above are clearly about students’ *perceptions* of how employers (and others) will view their degrees, the evidence we have cited previously suggests that labour market outcomes are indeed often related to place of study (e.g. Bathmaker et al. 2016), although also entangled with social class (Ingram and Allen 2019). However, while the majority of previous work in this area (at least that published in English) has tended to focus on the inequalities related to students in systems with a high degree of vertical stratification (such as the UK and US), our data suggest that students across Europe – even in much ‘flatter’ systems with much less significant status differences between institutions – also indicated that their ability to achieve their work-related aspirations may well be mediated by the place at which they were studying. Thus, while there may remain quite substantial differences in how national governments structure their HE systems and the importance they place on achieving strong vertical or reputational differentiation (Hazelkorn 2015), it appears that irrespective of whether nations are following the ‘neo-liberal model’ (in which resources are concentrated in a small number of elite institutions) or its ‘social-democratic’ counterpart (where all universities are supported to pursue high quality teaching and research) (Hazelkorn 2015), there is considerable homogeneity in the views of their students.

## The relevance of discipline

Students’ narratives about their aspirations for employment were also strongly inflected with assumptions about the subject they were studying. A considerable number of our focus group participants, from all six countries, spoke at length at what they perceived to be a lack of public understanding of ‘non-STEM’ subjects and assumptions that, if they were enrolled on an arts or social science degree, they would find it considerably more difficult to find a job on graduation:

I personally get the impression that other people, well, when I say: 'Hey, I’m studying politics and sociology!', they initially have absolutely no idea what I’m doing and what I’ll do at the end of it all (German focus group)

The majority of people, when I tell them I’m studying sociology, stop and say, ‘What kind of **** is that?’. They don't understand what I am studying and they think I’m here wasting my time, because they don't see any clear opportunities which might result from it. (Spanish focus group)

Indeed, a participant in one of the Spanish focus groups made a model of a stone to represent how she felt she was viewed by others. She then went on to explain:

I have made a stone, because sometimes I feel that [...] political sciences [...] it's as if [other people] didn't even know that the course existed. It's like a stone that's lying there and nobody sees it because the people don't know that it exists, nor what use it has; they ask me, ‘Do you want to be a politician?’ and I say, ‘No, I am studying politics, I don't want to be a politician,’ … 

While such comments were evident in all six nations (reflecting the common prioritisation of STEM in political debate, mentioned above), they were most common in Spain – likely because of the high levels of graduate unemployment at the time of our fieldwork (see discussion above) and participants’ serious concerns about whether they would be able to find a job on graduation.

Students’ views about the subjects they were studying were also informed, not just by the extent to which they believed they were ‘understood’ by wider society, but also by where they thought their subject stood relative to others in an ostensible hierarchy. Many students from all six nations tended to believe that, in general society, subjects were positioned hierarchically – largely on the basis of entry requirements, the status of the job to which they were linked and/or perceived earning potential. For example, participants spoke about how those studying law and medicine were held in particularly high regard:

I think the first time … when they [my family] realised I was a student of law, they saw me as someone who in the future would be the one wearing the suit, ‘That man, that demeanour, that pose’; and they see it all as something very superior, to have reached a height, to be a lawyer, the greatest of the great, ‘a fully formed, man’ … (Spanish focus group)

… if you study medicine they see you as a clever girl who studies a lot, who will be a good medic, who will contribute a lot, who will cure people. (Spanish focus group)

This can be contrasted with a discussion amongst social work students in one of the German focus groups in which they noted the low status that tended to be attached to their subject of study, and argued that this could be explained, to some extent at least, by the low status of the social work profession.

Students studying such vocational programmes also intimated, however, that the low status attached to their courses was also related to assumptions about their ‘applied’ and thus ‘less challenging’ nature. The German social work students described the kind of comments they received from others in the following way:

[People say] ‘I’m sure that's hard work too,’ but [they think] it's not the same as a super-challenging course with lots of … I mean, ‘It's really hard work and it's great, but it doesn't have the same standing.’ (German focus group)

Such statements resonate with scholarship on subject choices made at earlier stages of education, which has shown how those subjects associated with abstract reasoning are often viewed as harder and ‘masculine’ in nature, and thus of higher status (Mendick 2005).

While there was no evidence that such views had affected the aspirations of the students in our sample (including those of the social work students, discussed above), they were often marshalled as part of a broader critique of the value many social actors attributed to particular disciplines. Indeed, education students in some of the Spanish focus groups commented that they were often seen as ‘people who are not doing a serious degree’ but then went on to argue that the individuals who view them in this way ‘[do not] attach the correct amount of importance to the task of the educator within society’. This type of perspective was articulated most explicitly by students in one of the German focus groups:

I think that the purpose of university education in our society has adopted a purely economic … economic objective because we’re seeing that the humanities subjects are being scaled down and funding for the humanities has been drastically cut whereas the STEM subjects are being pushed hard and so only subjects that are economically relevant, important for a society, whilst other subjects aren't.

As noted previously, governments across the world have been trying to influence the aspirations of young people. In many nations, this has included various incentives to steer students away from the arts and humanities and into STEM disciplines – in the belief that the latter are of more societal benefit and lead to higher salaries. There is certainly evidence from our research across Europe that many students were aware of such initiatives – and also of public perceptions based on similar assumptions. However, we found little data to suggest that, despite this awareness, students regretted their choices. Indeed, many seemed to have been aware of these views before choosing their degree programme. This suggests, firstly, that students have rather different views about the purpose of HE than policymakers and other social actors. Although many were worried about their employment prospects, particularly if they were studying a subject that they believed to have low social status or visibility, this rarely affected how they valued their degree; they continued to hold non-economic attributes in high regard, even if they were aware they may not often be recognised by others. Secondly, policies that aim to shape students’ aspirations by pointing out salary differentials by subject may not be very effective in bringing about change given students’ already considerable awareness of putative differences by discipline, and their apparent prioritisation of other concerns when making subject choices.

## Conclusion

In our work on other aspects of European HE, we have tended to emphasise some enduring differences between countries highlighting, for example, variation by nation-state in the extent to which students are viewed as ‘mobile’ individuals (Brooks 2018a); significant political actors (Abrahams and Brooks 2019); consumers (Brooks and Abrahams 2020); and recipients of a public service (Lazetic 2019). Nevertheless, much more commonality across Europe is evident when we examine students’ aspirations for their lives post-graduation. As we have demonstrated above, students typically hoped that their HE would enable them to develop intellectually and personally, as well as facilitate a smooth transition into the labour market. While non-economic aspirations were articulated quite frequently, in all countries it was those that related to employment that tended to be foregrounded. This corresponds not only to the dominant emphasis within policy across Europe but also the primary focus of the academic literature in this area, discussed at the start of the article. Moreover, such data tend to support, at least to some extent, those who have argued that important commonalities are now evident across Europe, associated with the reconfiguration of European universities around an Anglo-American model (Cantwell and Slaughter 2012; Moutsios 2013).

However, while this could be read as evidence of the further instantiation of a ‘European learning space’ (Lawn 2009), our data indicate how aspirations were also sometimes shaped by particular national contexts. This is evident in, for example, the particular concern about labour market outcomes that emerged in the Spanish data, linked, we have suggested, to the high levels of youth unemployment that prevailed at the time of our data collection. It is also manifest in the greater emphasis placed on collective (rather than individual) aspirations, apparent in Denmark, Germany and Poland – the three countries where HE fees are still funded by the state and where, as a result, it is likely that discourses related to the importance of public education are more prevalent. Furthermore, we have shown how, within nation-states, many students indicated that they believed their ability to realise their aspirations might be affected by the institution they had attended, suggesting that the space of the campus can also serve to shape students’ views about the future.

Given the literature discussed previously, it is interesting to note that the majority of our respondents tended to believe strongly that the institution one attended had a significant influence on employment destinations, but made fewer comments about the impact of an individual's social class. As we noted above, although there are some studies that indicate that institutions can have a mediating effect on class – with students from low socio-economic groups who attend prestigious universities having an increased chance of entering well-paid, professional jobs (Bathmaker et al. 2016) – other work has shown the endurance of class differences among those attending the same type of institution (e.g. Hurst 2018). While it is possible that our participants’ views were informed by assumptions about social class – e.g. a belief that HEIs tend to have relatively class-homogeneous populations, and that this affects employment outcomes – the absence of references to any within-HEI variation was notable. This may be because students, in interview situations, often indicate that social class does not have any bearing on their own decisions, even when other evidence points to the contrary (see, for example, Armstrong and Hamilton 2013). It is possible, though, that such perspectives may also be influenced by our methods. In focus group situations, with peers from the same institution, it is perhaps easier for participants to reflect on the impact of what they have in common (a shared institutional affiliation) rather than what may divide them (potentially different socio-economic backgrounds). Nevertheless, irrespective of this limitation, the similarities across national contexts were substantial. Students’ perceptions about the likely impact of institution on their employment aspirations were not confined to only those in countries with strongly vertically-differentiated systems, where one might expect the rewards of HE to be more variable. Instead, it appeared that students in all countries believed that employers and other social actors made judgements based on institution of study, and that these could have a considerable effect on labour market transitions.

Finally, it is important to note that while the most commonly articulated aspirations were those relating to employment, our participants certainly did not define their HE experience solely in these terms; for the vast majority of the students we spoke to, a degree was significantly more than a qualification for the labour market (irrespective of whether they viewed themselves as ‘investors’ or ‘insurers’). This was evident in the range of non-job-related aspirations that they told us about and, perhaps most strikingly, in their cogent critique of the economic focus of government policy for HE, and what they perceived to be overly-narrow societal views – that often valorised courses linked to high status and well-paid careers and problematised those that had less obvious connections to particular jobs or that were associated with less well-remunerated professions. Here, we see a much more expansive understanding of HE than that which is implicit and often explicit in the pronouncements of policymakers across Europe.

## Notes

1 Skilled manual and unskilled occupations were classified as ‘working class’; all others were classified as ‘middle/upper class’.

2 The focus groups were translated by professional translators and, in a small number of cases, university students. The quality of the translation was checked by the person who conducted the focus group and/or members of the project team.
